# Recent Advances in Probing Electron Delocalization in Conjugated Molecules by Attached Infrared Reporter Groups for Energy Conversion and Storage

**DOI:** 10.1021/acsaem.4c03246

**Published:** 2025-02-06

**Authors:** Deepak Devadiga, Juchao Yan, Dheeraj Devadiga

**Affiliations:** † Department of Physical Sciences, Eastern New Mexico University, Portales, New Mexico 88130, United States; ‡ Manipal Technologies Limited, Manipal, 576104, Karnataka, India

**Keywords:** Vibration, electron delocalization, infrared probe, frequency, organic solar cells

## Abstract

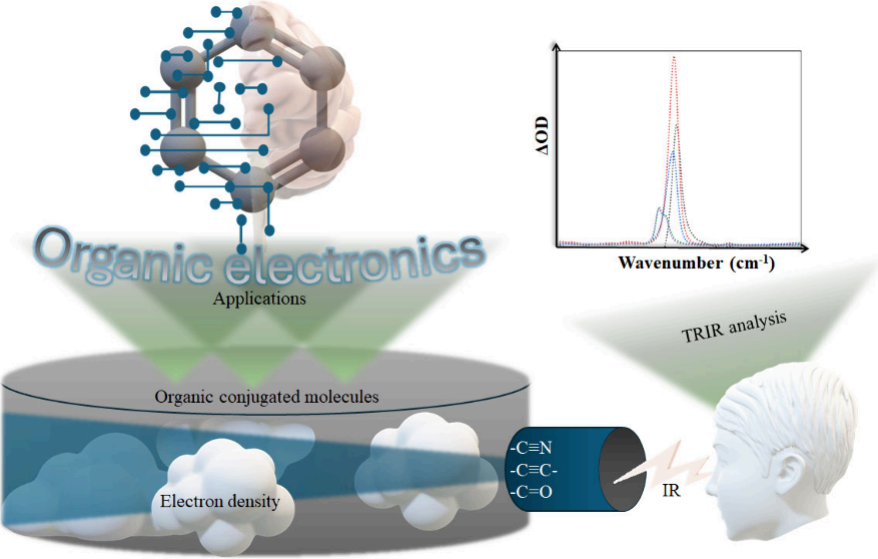

This review article reports an overview of the recent developments in the field of electron delocalization study in organic conjugated molecules by utilizing the vibration frequencies exhibited by the attached functional groups such as nitrile (−C≡N), alkyne (−C≡C−), or carbonyl (−C=O). A brief introduction to electron delocalization, methods for study, and their importance is given first, followed by the application of infrared spectroscopy in organic molecules. Details of molecules with various infrared reporter groups have been explained in respective subsections based on the functional groups. All the reported organic molecules have been structured and presented with the electron delocalization properties studied using an infrared reporter group. Finally, an outlook on this recently promising, exciting, and interesting field of probing electron delocalization using infrared reporter groups is provided.

## 1 Introduction

In 1994, the IUPAC defined electron delocalization as a quantum mechanical concept used in organic chemistry to study and understand the π-bonding in conjugated organic molecules.^1^ Usually, this type of π-bonding is not between the two atoms in the systems but bonding between each atom with a fractional double bond. Electron delocalization in the organic molecules can be studied by understanding the energy difference between the proposed molecular system (precisely localized single and double bonds) and the delocalized system.^2^ Moreover, this type of change is mostly observed in aromatic molecular systems where an empty π-orbital or a lone pair of electrons is conjugated with neighboring double bonds in the molecule.^3^

The electron delocalization concept plays a crucial role in chemical and biological sciences.^4,5^ The stability,^6^ acidity,^7^ and reactivity site^8^ of aromatic molecules can be explained through electron delocalization. Additionally, electron delocalization in the π-conjugated systems greatly influences the optical properties (absorption, emission, and band gap), electronic properties (hole and electron mobilities), and thermal properties.^9−12^ Also, electron delocalization stabilizes the intermediate states of the molecule and facilitates redox reactions, thereby allowing interaction sites in the molecule to be studied for particular biological systems.^13,14^ Therefore, molecules with π-conjugation with electron delocalization can be useful in the field of electronics, sensors, and optoelectronics.^15−17^

Electron delocalization has wide-ranging applications in energy conversion and storage. It increases the electrical conductivity of materials,^18^ which is essential for efficient charge transfer in fuel cells.^19−21^ It stabilizes the redox states of charged species,^22^ which is critical for storing and releasing electrons in battery materials.^23−27^ It lowers the energy gaps of conjugated organic molecules,^28^ which is indispensable for facile electron excitation and light absorption in advanced organic electronics,^29−31^ including light-emitting diodes, field-effect transistors, and organic solar cells (OSCs). It also enhances the catalytic activity and selectivity of active sites of catalysts,^32−37^ which is crucial for the target chemical reactions.

It is very important to understand the charge carrier properties of energy conversion and storage devices. Therefore, to completely examine the charge carrier dynamics in the molecules, various experimental methodologies have been made, such as transient photoconductivity (TPC),^38^ electrochemical impedance spectroscopy (EIS),^39,40^ steady-state photoluminescence (PL),^41^ transient absorption spectroscopy (TAS),^42^ time-resolved terahertz spectroscopy (TRTS),^43^ ultrafast pump–probe spectroscopy,^44^ and time-resolved photoluminescence (TRPL).^45^ Further insights were obtained through theoretical methods, especially Monte Carlo simulations^45^ and density functional theory (DFT).^46^ The combination of both experimental and theoretical investigations is vital in understanding the design strategies and underlying properties to improve the performance of the devices.

It is important to correlate the structural features of the molecule with the electronic properties. Therefore, ultrafast spectroscopy techniques have been developed and used to study the molecules in the terahertz (THz), visible (Vis), and near-infrared (NIR) spectral ranges.^47^ These techniques demonstrated charge carrier generation and their distribution in the molecules, followed by the effect of composition, processing conditions, and morphology on their electronic properties. Recently, this method of study was implemented in the lead-halide perovskites, which were processed through solution state, and results revealed that it has long carrier diffusion lengths which implied that their electrical properties could be affected by their distinct architectures.^48^

One of the sensitive preliminary characterization techniques for organic molecules is vibrational spectroscopy. Along with multidimensional analysis, time-resolved infrared (TR-IR) spectroscopy was a very exciting tool for understanding the behavior of molecular charges and their excited states in their condensed phases.^49−52^ Where the study uses the absorption of functionalities of the molecules such as nitrile (−C≡N), alkyne (−C≡C−), or carbonyl (−C=O) as probes to quantitatively study electric fields in an organic system by utilizing the vibrational Stark effect (VSE).^53−57^ Also, fluctuations in the infrared absorption frequency of the functional groups due to the external changes shed light on the noncovalent interactions, solvation, and structural modifications. Therefore, this review aims to take a critical look at the literature and summarize the recent advances in probing electron delocalization in conjugated molecules by attached infrared reporter groups such as −C≡N, −C≡C–, or −C=O in order to improve the knowledge regarding electron delocalization in a research laboratory. Also, this review provides the possible future direction of this technique and states how structure-to-device performance relationships can be achieved.

## 2 Molecules with Nitrile (−C≡N) Functionality

Nitriles are the general name for common organic molecules comprising one or more nitrile functional groups. Nitrile functionality (−C≡N) comprises carbon and nitrogen atoms linked through the triple bond.^58^ Molecules with nitrile functional groups are polar and exhibit high dipole moments.^59^ In addition to the nitrile-moiety-containing organic molecules, the nitrile moiety is also present in some inorganic compounds like cyanides.^60^ The nitrile functional group plays a crucial role in various applications of organic molecules such as solvents, displays, OSCs, perovskite solar cells (PSCs), organic light emitting diodes, supercapacitors, sensors, drug delivery systems, the rubber industry, and the pharmaceutical industry.^61,62^

### 2.1 –C≡N Functionalities as the IR Probe for the Electron Delocalization Study

The −C≡N stretching band is often easily observed around 2200–2300 cm^–1^.^63^ However, conjugation in the molecules lowers the band of aromatic nitriles compared to saturated nitriles.^64^ In this section, we will review the electron delocalization study in the molecules by utilizing the nitrile functionality as the IR probe.

**Figure 1:**
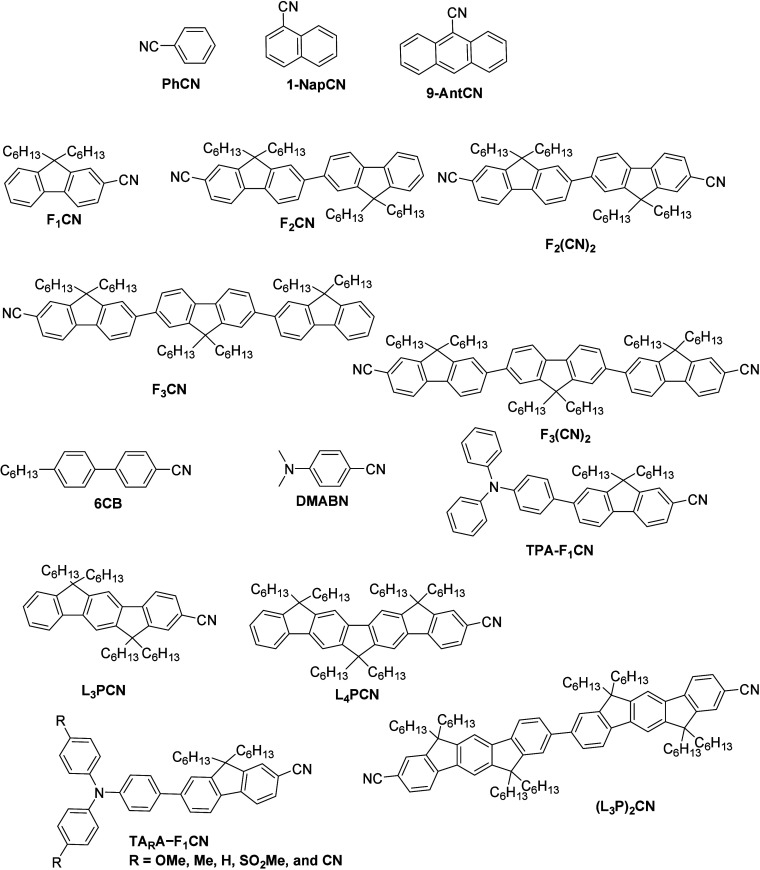
Structure of the molecules studied by Mani et al.^65−68^

**Figure 2:**
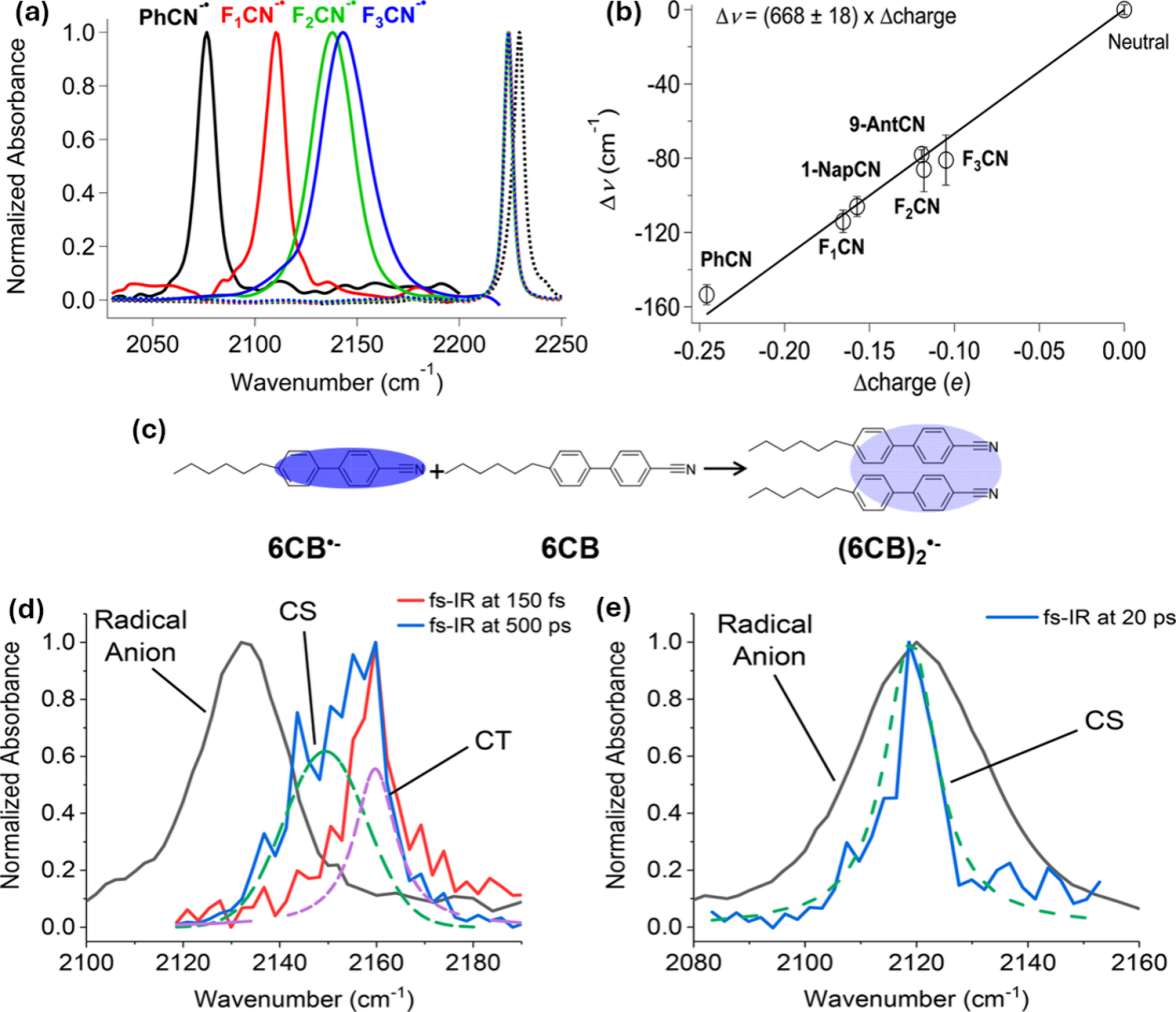
(a) Dotted lines represent the FTIR spectra of neutral molecules in THF solution (**PhCN**, **F**_**1**_**CN**, **F**_**2**_**CN**, and **F**_**3**_**CN**), and solid lines represent TR-IR spectra of the molecules in free radical anions form obtained ∼50 ns to 1 μs after pulse radiolysis. (b) The calibration curve plotted by Mani et al.^65^ using experimentally observed changes in the IR absorbance frequency of nitrile functionality (Δν) and the calculated changes in charges from electrostatic potentials using a grid of the nitrile functionality between the neutral and anion states (Δcharge). Reproduced from ref (65). Copyright 2015 American Chemical Society. (c) Intermolecular electron delocalization in a dimer radical anion of **6CB** molecules and the parallel conformation of the dimer. Reproduced from ref (66). Copyright 2017 American Chemical Society. (d,e) Generation of electric field observed in the CT and CS states of **TPA-F**_**1**_**CN** molecule probed by fs-IR after 400 nm photoexcitation in the ν(−C≡N) region. IR spectra of the **TPA-F**_**1**_**CN** molecule in THF (d) and DMF solvent (e). The green and purple dotted lines represent the CS and CT spectra, respectively. The black line represents the TR-IR spectra of the free radical anions of the **TPA-F**_**1**_**CN** molecule obtained ∼4 μs after pulse radiolysis. Reproduced from ref (67). Copyright 2018 American Chemical Society.

In 2015, Mani et al.^65^ studied the effect of the degree of delocalization of electrons to the ν(−C≡N) nitrile vibration in anions of nitrile-substituted oligofluorenes. Initially, the authors observed the shifting IR frequencies in the anion radicals of the phenyl-ring-based molecules (**PhCN**, **1-NapCN**, and **9-AntCN**) and fluorene-based molecules (**F**_**1**_**CN**, **F**_**2**_**CN**, and **F**_**3**_**CN**). The structures of the studied molecules are depicted in Figure 1. Their study revealed that as the number of benzene rings increases, the difference between the neutral molecule and anion (Δν) becomes smaller, as represented in Figure 2a. Moreover, using a series of mononitrile-functionalized molecules, the authors plotted the calibration curve by noting shifts in −C≡N frequencies to understand the changes in the electronic density distributions in those molecules (Figure 2b). The aryl to which the −C≡N is connected determined the line widths for the −C≡N IR absorptions in the anions used in that study, which varied by a factor of 5. Moreover, the line widths were found to be sensitive to flexible dihedral angles; however, the authors failed to shed light on these. Therefore, the authors stated that as their comprehension grows, they will provide insightful information about the dynamic movements in the structures they are connected to. The authors also used a moment-based technique to provide a more precise image of the anion’s delocalization states. The authors established a novel technique (IR-CHARGE) to investigate the exchanging electron behavior in the anions of dinitrile-functionalized oligofluorenes in combination with existing theory, which are categorized as organic mixed-valence compounds. Their findings demonstrated that electron localization is not always static but can instead be dynamic. In these mixed-valence compounds, physical electron localization, as measured by TR-IR, reveals a symmetry breakdown where the charge centers and bridge are strongly connected. The authors were unable to make it clear whether symmetry was broken after or before the addition of an electron, but an equilibrium is rapidly reached for a minimal activation energy of < *k*_B_*T* (where “*k*_B_” is the Boltzmann constant and “*T*” is the absolute temperature in Kelvin). The dihedral angles observed between the fluorene units were attributed to the reduced activation energy. The presence of dihedral angles in polymers is likely to result in a comparable energy barrier, which can determine the limit of electron transport in molecular wires. The calculated energy barrier supports the idea that flaws of this kind might impair the transport capabilities, as it is around 2–4 times less than those associated with “bad” dihedral angles.

Furthermore, additional evidence of the exceptional sensitivity of the vibration frequency of nitrile to the degree of electron delocalization in the anion state has been reported by the same group by using a room-temperature liquid crystal molecule, i.e., 4-*n*-hexyl-4′-cyanobiphenyl (**6CB**, Figure 1).^66^ In the first part of their study, the authors illustrated that the sensitivity of the nitrile functional group vibration was mostly an intrinsic property of the nitrile moiety and does not depend on the polarity of the solvent in which it was dissolved. Therefore, the authors concluded that in order to investigate the extent of electron delocalization in a variety of organic systems calibration curves that link the IR shift of nitrile functionality with the variations in the electronic density distribution from the neutral state to anionic state can be employed. In the second part of the study, the authors used the sensitivity of the nitrile functionality to study the formation of the dimer radical anion of **6CB** molecules at room temperature. IR shifts of the vibration corresponding to nitrile functionality clearly demonstrated that the two molecular fragments experience an equal distribution of extra electron charge. Their infrared research could potentially clarify some aspects of the dimer radical anion’s electronic structure when paired with traditional electronic absorption spectroscopy and electronic structure predictions. At last, the investigators deduced that dimerization of the radical anions of **6CB** is significantly influenced by the existence of a long alkyl chain and the establishment of π–π interactions (Figure 2c).

Later, the same group in another study found that the nitrile vibration of the charge-separated (CS) state of 4-(dimethylamino)benzonitrile (**DMABN**, Figure 1) experienced a significant blue shift in frequency when compared to that of the radical anion of benzonitrile (**PhCN**) molecule in a tetrahydrofuran (THF) solvent.^67^ This was demonstrated by the authors using TR-IR spectroscopy, following laser flash photolysis and pulse radiolysis. This finding confirmed that there is an electric field in the CS state and allowed the authors to measure its intensity inside the VSE framework. The concept of a twisted intramolecular charge-transfer (ICT) state is made more compelling by the newly acquired information. The authors further discussed that this type of shift in the vibrational frequency of nitrile functionality exists in another structurally similar molecule comprising triphenylamine and fluorene units with nitrile functionality (**TPA-F**_**1**_**CN**, Figure 1). By probing the fs-IR in the ν(−C≡N) region after 400 nm photoexcitation, the authors noted the generation of the electric field in the charge-transfer (CT) and CS states of **TPA-F**_**1**_**CN** molecules (Figure 2d,e). Due to dielectric screening, the ν(−C≡N) frequency of the CS state in a polar solvent is the same as that of the free radical anion. The study’s findings demonstrated that while tracking photoinduced charge transfer processes using ν(−C≡N) and maybe other vibrational modes, the VSE must be taken into account. The authors’ work has shown that nitrile vibration has exceptional sensitivity, making it useful for studying photoinduced ICT processes. It could be used to measure the induced electric field and determine the kind of electron delocalization.

**Figure 3:**
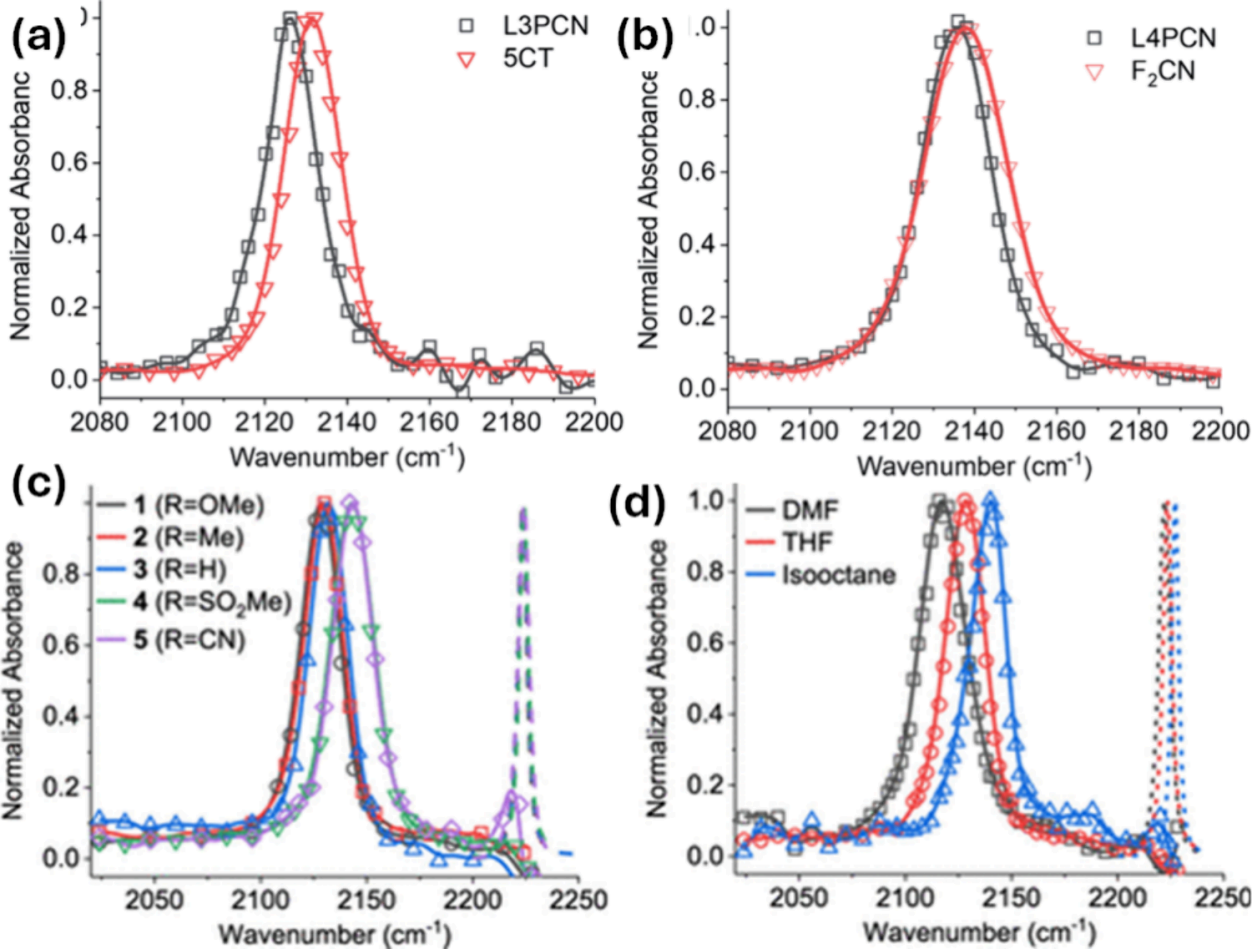
(a, b) TR-IR spectra in THF solvent. (a) L_3_PCN̅̇ and 5CT̅̇. (b) L_4_PCN̅̇ and F_2_CN̅̇. These spectra were recorded ∼100 ns after pulse radiolysis of neutral solutions. Reproduced from ref (68). Available under a CC BY-NC 3.0 license. Copyright 2021 The Royal Society of Chemistry. (c, d) FTIR spectra of the neutral forms of the **TA**_**R**_**A–F**_**1**_**CN**, and TR-IR spectra of their free anionic forms, were obtained ∼50 to 500 ns after pulse radiolysis. (c) Molecules in THF solution; (d) **TA**_**OME**_**A–F**_**1**_**CN** in DMF, THF, and isooctane solvents. Reproduced from ref (71). Copyright 2020 American Chemical Society.

Moreover, the line widths and extinction coefficients of the ν(−C≡N) band of the neutral and radical anions forms of oligo(*p*-phenylene)s comprising nitrile functionality (**L**_**3**_**PCN**, **L**_**4**_**PCN**, and **(L**_**3**_**P)**_**2**_**CN**) have recently been determined in another work conducted by the same group.^69^ The structures of the studied molecules are depicted in Figure 1. The study revealed that the information on IR intensities and line widths indicated the −C≡N vibration was related to the electronic and nuclear structural changes, even though the ν(−C≡N) band was a localized vibration. Variations in the IR line widths and intensities reflected this coupling, which is more pronounced in the charged species. The authors clearly observed an enhancement in the ν(−C≡N) absorption frequency, with one of the molecules exhibiting the most intense ν(−C≡N) absorption band reported to that date. Using ladder-type oligo(*p*-phenylene)s comprising nitrile functionality, the authors have shown that the line width of nitrile vibration can report on electronic and nuclear variations linked with the flexible dihedral angle movements only in cases where a charge has scope to move in space. Interestingly, the authors observed the same line widths for the shorter molecules despite the absence or presence of flexible dihedral angles, e.g., **L**_**3**_**PCN**^•–^ vs **5CT^•^®** (Figure 3a). Conversely, the suppressed line width broadenings are noted in the longer molecules when the authors removed the flexible dihedral angles. For example, the authors observed a narrower full-width half-maximum of 19.4 cm^–1^ for the **L**_**4**_**PCN**^•^® molecule compared to that of 24.1 cm^–1^ for **F**_**2**_**CN**^•^® (Figure 3b). The results of this study provided credence to the theory that charges in conjugated chains are dynamic and subject to variations in charge density caused by vibrations of the molecules.^70,71^ Additionally, their work lays the groundwork for the utilization of intensity and line width data as well as IR frequency shifts as parameters of infrared probes for charges.

Another study by Mani et al.^68^ revealed the nature of the excess electrons in the donor–acceptor (D–A) molecules by changing the substituents attached to the molecules (**TA**_**R**_**A–F**_**1**_**CN**, where R = −OMe, −Me, −H, −SO_2_Me, and −C≡N) and the solvent polarity. During the study, the authors observed that the nitrile ν(−C≡N) vibrations in radical anions of these synthesized molecules were affected by the electron push–pull capability of the substituents attached to the amine donor unit and solvation in various solvents with different polarities (Figure 3c, d). Furthermore, it was demonstrated by quantum computations that the push–pull capacity is capable of translating the position of an excess electron while maintaining a relatively constant width. In contrast, solvation modifies both, increasing the electron’s compactness in polar solvents. Thus, their research suggested that solvation is more important in regulating the type of excess electrons, and additional tuning is made possible by a synthetic modification that affects electron push–pull capabilities.

**Figure 4:**
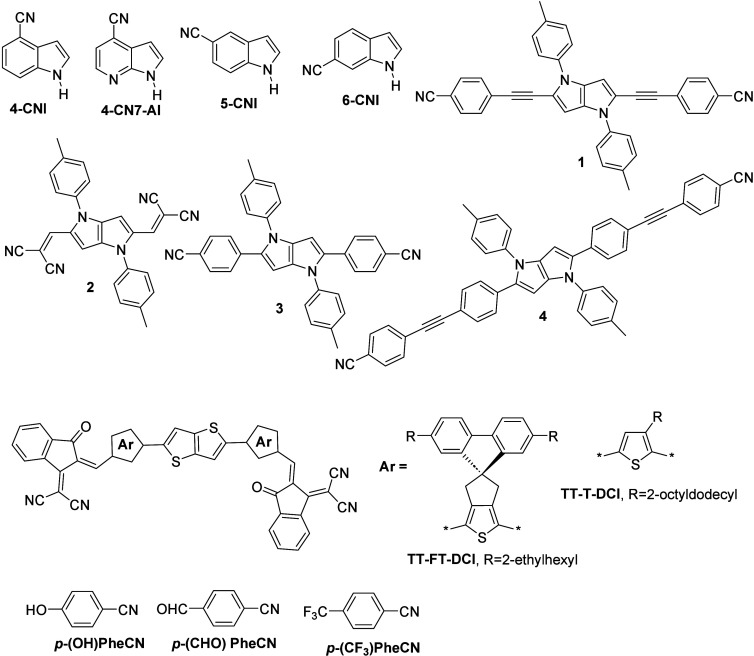
Structure of the molecules studied by Choi et al.,^72^ Liu et al.,^73^ Yang et al.,^77^ Verma et al.,^90^ and Jinnai et al.^93^

In addition to **PhCN** and **DMABN** molecules, Choi et al.^72^ investigated other *para*-substituted benzonitrile probes, such as ***p*****-(OH)PheCN**, ***p*****-(CF**_**3**_**)PheCN**, and ***p*****-(CHO) PheCN** (Figure 4). In that study, the authors compared the effect of electron-donating/withdrawing functionality on the extinction coefficient as well as the vibrational lifetime of the −C≡N probe. Results revealed that the **DMABN** molecule exhibited the best IR probe when compared to the other four molecules. The strong electron-donating ability of the dimethylamine functional group pushes the electron density toward the nitrile group, which induces the high molar extinction coefficient of the −C≡N stretching vibration, and they have noted the longest vibrational lifetime for the same among other samples.

**Figure 5:**
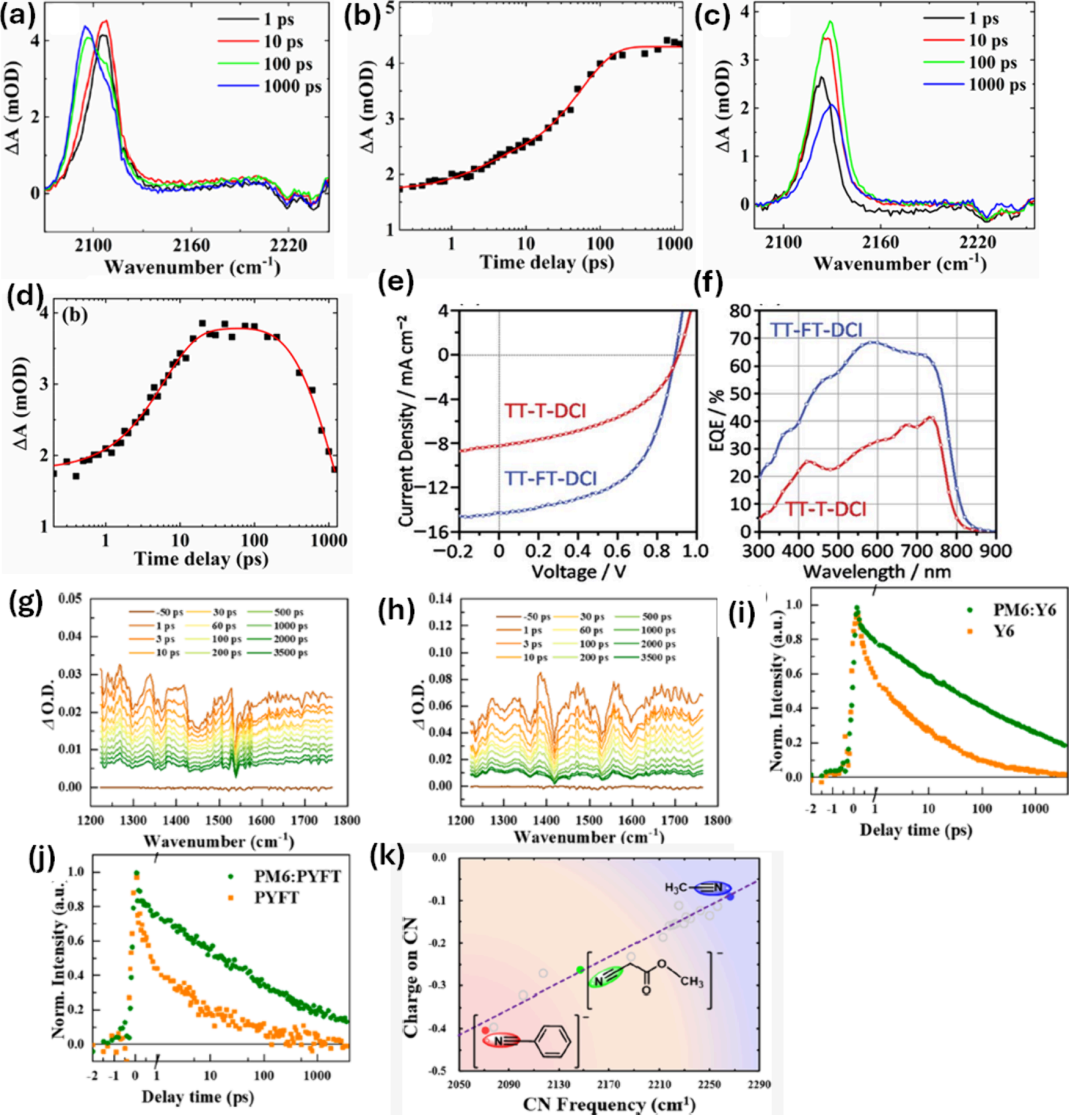
(a) TA spectra were obtained for **4-CNI** in IPA at representative time delays. (b) TA kinetics obtained for **4-CNI** in IPA at 2095 cm^–1^. (c) TA spectra were obtained for **4-CN7AI** in IPA at representative time delays. (d) TA kinetics obtained for **4-CN7AI** in IPA at 2129 cm^–1^. Reproduced from ref (73). Copyright 2022 American Chemical Society. (e) *J*–*V* characteristics of the **PBDB-T**:**TT–FT–DCI** and **PBDB-T**:**TT–T–DCI**-based OSC devices and (f) their external quantum efficiency spectra. Reproduced from ref (93). Available under a CC BY 3.0 license. Copyright 2022 The Royal Society of Chemistry. (g) Transient IR spectra of **PM6**:**Y6** D/A heterojunction films at selected delay times. (h) **PM6**:**PYFT** D/A heterojunction films at selected delay times. (i) Kinetic traces monitoring at 1400 cm^–1^ for **PM6**:**Y6** and **Y6** films. (j) Kinetic traces monitoring at 1400 cm^–1^ for **PM6**:**PYFT** and **PYFT** films. Reproduced from ref (94). Copyright 2022 American Chemical Society. (k) The graph of ν(−C≡N) value (experimentally determined) and the q(−C≡N) value (computationally determined the grid fitting method) for the compounds. Reproduced from ref (77). Copyright 2023 American Chemical Society.

Liu et al.^73^ procured 4-cyanoindole (**4-CNI**, Figure 4) and 4-cyano-7-azaindole (**4-CN7AI**, Figure 4) molecules and studied the variation in intensity, ε(−C≡N), and frequency, ν(−C≡N), of the −C≡N stretching vibrational bands upon electronic excitation in a series of solvents such as dimethyl sulfoxide (DMSO), 1,4-dioxane (Dio), isopropanol (IPA), trifluoroethanol (TFE), and ethanol (EtOH). The authors reported that in IPA solvent, the −C≡N stretching vibrational frequency of **4-CNI** and **4-CN7AI** molecules in the excited state was red-shifted by >110 and 100 cm^–1^, respectively, from its ground state values, and also noted the increase in their intensity by a factor of 13.5 and 12.6, respectively. The results of the UV_pump_–IR_probe_ analysis for **4-CNI** and **4-CN7AI** molecules in IPA solvent were depicted in Figure 5a–d, measured with a pump wavelength of 315 and 320 nm, respectively. In addition, the authors observed that the effects on ε(−C≡N) and ν(−C≡N) due to solvent relaxation in the excited state are different; although this process usually increases ε(−C≡N), ν(−C≡N) either increases, decreases, or remains the same depending on the solvent and the solute. Their findings demonstrated that there are two ways in which solvent relaxation might impact the stretching vibration of the nitrile functionality. One effect is to increase the electronic conjugation between the aromatic ring and nitrile functionality, which is independent of the solute–solvent system and leads to a decrease in the frequency and an increase in the intensities of the −C≡N stretching vibrations. The other effect is to modify the −C≡N group’s local environment, which can alter the frequency and intensity of the bands depending on the solute–solvent system. Lastly, these findings directly corroborated the idea that the stretching vibration of the nitrile functionality can be used as a sensitive infrared sensor to study the dynamics and mechanisms of the charge transfer process in the aromatic nitrile molecules.^67,74^

Chappell et al.^75^ used nineteen 7,7,8,8-tetracyanoquinodimethane salts to study the dependence of the −C≡N stretching frequencies on their respective degrees of charge transfer. They found the linear dependence between them. Also, Juchnovski et al.^76^ observed the linear correlation between the −C≡N stretching frequencies of α,β-diaryl cyanoethylene derivatives with the −C≡N bond orders calculated using Hückel molecular orbital model. Based on these findings, Yang et al.^77^ studied the relationship between −C≡N stretching frequencies and total charge in the nitrile group, q(−C≡N). Initially, the authors used mononitrile molecules whose stretching vibrations of −C≡N are extended over a frequency range of ca. 200 cm^–1^. Then, by using DFT analysis, the authors determined the net charges on those molecules under various conditions such as in gas or solution state, neutral or anion form, and in solvents with different polarity. Results revealed the linear relationship with a slope of about 637 ± 30 cm^–1^/charge between the stretching frequencies and the total charge on the −C≡N (Figure 5k). From this result, the authors quantified the degree of charge transfer in the lowest excited singlet electronic states of two indole-based molecules, such as 5-cyanoindole (**5CNI**) and 6-cyanoindole (**6CNI**). They also concluded that, in a variety of applications, this linear correlation function can be used as a practical scale to determine the degree of charge separation.

Many acceptor–donor–acceptor (A–D–A) type molecules have been observed to experience the excited-state symmetry breaking (ES-SB) process. During this phase, the electronic excitation, which was earlier dispersed throughout the entire molecule, at least partially localizes on one side.^78−80^ This was first indicated by the solvatochromic property study of two-branched dye molecules that were as large as of the molecules with a single D–A branch.^81−87^ TR-IR was then used to gather clear proof by tracking vibrations that are IR inactive in the symmetric quadrupolar form and turn into IR active upon ES-SB.^88,89^

Moreover, Verma et al.^90^ examined the accessibility of interbranch coupling and the solvation energy by using stationary absorption and emission analysis. Moreover, they have proposed the use of a time-resolved vibrational spectroscopy technique for instantly recognizable evidence for the ES-SB process in dye molecules (**1** and **2**, Figure 4). In their study, they prepared two A–D–A dye molecules with cyanophenyl or dicyanovinyl accepting groups. Despite the weaker electron-accepting properties of the cyanophenyl functionality compared to dicyanovinyl moiety, the authors observed the ES-SB process in the cyanophenyl-based dye molecule even in weakly polar solvent systems; at the same time, the excited state of the dicyanovinyl-based dye molecules remained symmetric even in the solvents of high polarity. Their study results underscore the impact of the coupling between the two A–D branches of the molecule in resolving a propensity of the quadrupolar dye molecule for the ES-SB phenomenon and becoming dipolar. For the symmetry-breaking phenomenon to occur, the coupling energy must be lost, which can be offset by a gain in solvation energy. In the case of another dye molecule **2**, the substantial coupling could not be counterbalanced by solvation energy. This substantial coupling was attributed to the small branches of the molecule. Since the cyanophenyl-based dye molecule has longer D–A branches, it encountered weaker interbranch coupling even though it was a weaker electron-accepting group. However, their comparison with the other A–D–A based on cyanophenyl molecules (**3** and **4**, Figure 4) of larger D–A distances^91,92^ does not result in small coupling, specifically when ethynyl functionalities were used as spacers.

Jinnai et al.^93^ discussed the effects of sterically bulky and rigid molecular structures of nonfused nonfullerene acceptors on transient photon-to-current dynamics. For this purpose, the authors synthesized π-conjugated molecules, **TT–FT–DCI** and **TT–T–DCI** (Figure 4). Their photophysical properties and the theoretical calculations revealed that the introduction of the 2,7-bis(2-ethylhexyl)fluorene unit efficiently rigidified the backbone of the π-conjugated molecule. Moreover, they have used those molecules for OSC application and found that devices based on **PBDB-T**:**TT–FT–DCI** exhibited improved short-circuit current density and fill factor compared to the devices based on **PBDB-T**:**TT–T–DCI**; therefore, it exhibited a PCE of about 7.13%. The current density–voltage (*J*–*V*) characteristics of the prepared devices and their external quantum efficiency spectra are depicted in Figure 5e and Figure 5f, respectively. In their research, a TR-IR absorption spectroscopy study revealed the pristine thin films of molecules comprising the 2,7-bis(2-ethylhexyl)fluorene unit exhibited smaller conformational relaxation when compared to that of the thiophene-based molecule under photoexcitation conditions. Furthermore, the **TT–FT–DCI** molecule exhibited longer lifetimes of the excited and CS states in the blend films than the **TT–T–DCI** molecule. Also, for **PBDB-T**:**TT–FT–DCI** films, the flash-photolysis time-resolved microwave conductivity measurement showed a longer lifetime of free carriers. Additionally, near the ZnO electrode surface, **PBDB-T**:**TT–FT–DCI** adopted a face-on conformation in the CS state. This face-on conformation was attributed to the sterically bulky 2,7-bis(2-ethylhexyl)fluorene units in the **TT–FT–DCI** molecules. Finally, the authors concluded that efficient molecular design for the proper kinetics of photocurrent generation can be achievable by employing the stiff π-conjugated backbone with inhibited self-aggregation of the nonfused acceptor.

**Figure 6:**
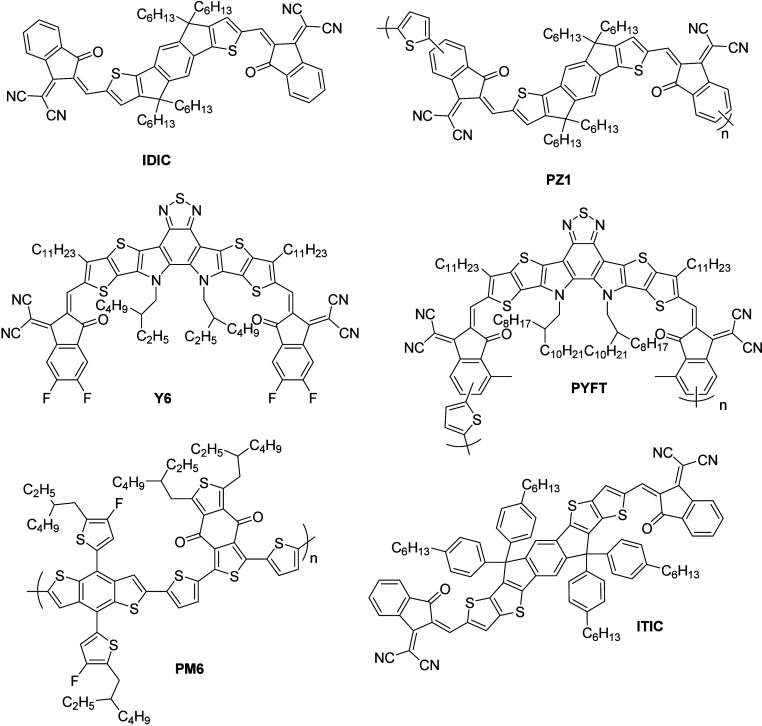
Structure of the molecules studied by Zhang et al.^94^ and Song et al.^95^

Transient mid-IR spectroscopy was used by Zhang et al.^94^ to observe the photogeneration of free carriers in solid films of **Y6**, **IDIC**, **PYFT**, and **PZ1** molecules (Figure 6). Moreover, to understand the free carrier distribution in the excited state of the molecules, the authors investigated the vibrational behavior in the excited state. They observed a higher free carrier concentration in the polymerized acceptors **PYFT** and **PZ1** than in their respective small-molecule acceptors **Y6** and **IDIC**, and they attributed this to the reduced exciton binding energies after polymerization. In addition, the lifetimes of photogenerated free carriers of these molecules were on the order of hundreds of picoseconds, and these values are much shorter when compared to that of the inorganic semiconductors. Furthermore, the authors prepared the D/A heterojunction using **PM6**:**Y6** and **PM6**:**PYFT**, and it displayed higher free carrier concentration with a longer lifetime of over 3 ns owing to the charge transfer process. Transient IR spectra of the prepared D/A heterojunction and their kinetic traces monitoring at 1400 cm^–1^ are depicted in Figure 5g–j. Besides, the authors performed the DFT calculation, and the study revealed that the decrease in the exciton binding energy of polymerized samples was due to the decreased Coulombic interaction, which was induced by the increased delocalization of the holes/electrons after polymerization. Based on this study, the authors concluded that polymerization is one of the effective approaches to decrease the exciton binding energy of small-molecule acceptors, which will play a vital role in improving the efficiency of the OSCs.

Multispectral 2D spectroscopy with visible and mid-IR probes was used by Song et al.^95^ to understand the charge separation in an archetypal nonfullerene acceptors blend heterojunction formed by **PM6** and **ITIC** molecules. The structures of the **PM6** and **ITIC** molecules are depicted in Figure 6. Moreover, rapid electron transfer from the **PM6** system to **ITIC** molecules and hole transfer from the **ITIC** system to **PM6** molecules was revealed by analyzing the systems using two-dimensional electronic–vibrational spectroscopy and two-dimensional electronic spectroscopy, respectively. A comprehensive examination of two-dimensional electronic–vibrational spectroscopy analysis data showed that bound polaron pairs in the **ITIC** system are vital precursors for the charge separation process through hole transfer and are converted efficiently to delocalized polarons at the heterojunction between **PM6** and **ITIC** molecules on a time scale of nearly 100 fs. Nevertheless, bound polaron pairs in the **PM6** system undergo relaxation quickly to the ground state, and therefore, localized excitons played a key role as the main precursor for the charge separation process through electron transfer. Also, the rapid charge separation process at a small HOMO offset was attributed to the weak Coulombic interaction in the bound polaron pairs and the delocalization of polarons. Through this study, the authors highlighted the importance of the synergistic effect of bound polaron pairs and delocalization. Also, the study provided an important approach for the charge separation efficiency optimization in organic molecule-based D–A blend heterojunctions with a low potential offset.

The above section systematically demonstrates previous literature on using the −C≡N stretching vibration frequency as a tool for studying and quantifying charge separation dynamics, q(−C≡N), in organic molecular systems based on their structures, i.e., started with various simple phenyl rings, fused phenyl rings, biphenyls, planar fluorenes, nonplanar fluorenes, triphenylamine connected fluorenes, D–A type molecules, indoles, and finally the D/A heterojunctions.

### 2.2 The Study Outputs

- Previous study results revealed and established ν(−C≡N) as a reporter of the charged state and the charge transfer dynamics. Specifically, work from Yang et al.^77^ revealed that q(−C≡N) can be evaluated quantitatively using ν(−C≡N) through a linear relationship. Moreover, their research computed q(−C≡N) for various mononitrile-based molecules, revealing that ν(−C≡N) values vary linearly with q(−C≡N) values across a 200 cm^–1^ spectral range. This linear correlation between ν(−C≡N) and q(−C≡N) can be anticipated as a general method for charge separation study, making it pertinent in various fields of chemical and biological sciences.
- The previous studies indicate that there is a significant influence of q(−C≡N) on the ε(−C≡N). For instance, when compared to neutral nitrile species, their charged counterparts can exhibit a greater order of magnitude values of ε(−C≡N).
- Research by Choi and co-workers^72^ shows that in molecules bearing *p*-substituted nitriles, higher ε(−C≡N) values are noted when molecules have stronger electron-donating substituents. It also highlighted the role of conjugation in charge separation dynamics.
- Research from Liu et al.^73^ revealed that upon excitation of nitrile derivatives, molecules will experience an increase in ε(−C≡N) values attributed to photoinduced charge transfer, which further enhances the density of electrons on the nitrile functional group.
- The stretching vibration of −C≡N is an effectual probe for perusing locally excited (LE) states as well as ICT states, particularly **DMABN** molecules and organic photovoltaic (OPV) devices.
- The studies highlighted how charge distribution in the molecule can become localized because of ES-SB under specified conditions. For instance, investigation on A–D–A-based conjugated molecules shows that even though it was functionalized with a strong electron-withdrawing dicyanovinyl, it does not cause ES-SB in solvents of high polarity owing to substantial electronic coupling.
- Research on the nitrile-containing OPV acceptor, i.e., **Y6**, indicates that the stretching frequency of −C≡N remains constant across solvents of different polarity in its excited state, implying that ES-SB may not play a crucial role in achieving high PCE.
- The research revealed that charge separation as well as electron transfer in a prepared **PM6**/**Y6** blend occurs on a time scale of less than 230 fs, which indicates the quick dynamics in the process of charge transfer.
- Also, a broad positive peak in transient absorption spectra revealed that electron wave function delocalization occurs over multiple **Y6** molecules, which further enhances the charge generation efficiency.
- The stretching vibration of −C≡N functionality is used to evaluate the charge dynamics in OPV blends, and this method has potential applications in assessing the generated photovoltage by metal–insulator–semiconductor junctions through the VSE. Overall, the recent findings emphasize the usage of −C≡N stretching vibrations as charge transfer dynamics reporters, with implications for the design, synthesis, and optimization of PV materials.

## 3 Molecules with Alkyne (−C≡C−) Functionality

The alkyne (−C≡C−) functionality is a significant synthetic building blocks in organic chemistry. Alkyne functionality was also found in several natural products.^96,97^ Derivatives of conjugated enynes, comprising the 1,3-enyne backbone, can be found in numerous bioactive molecules^98,99^ and are significant intermediates in the synthesis route of the organic compounds and macromolecules.^100−103^ There are two types of alkynes: terminal and internal. Both the terminal C–H and the C–C bonds are reactive sites that can undergo numerous transformations.^104^ Besides, the terminal alkyne can easily undergo cycloaddition reaction, which enables the covalent bond formation between two chemical fragments to form molecules with complex molecular frameworks and for those molecules to find applications in cell biology, pharmaceutical, and material sciences.^105−107^

### 3.1 –C≡C– Functionalities as the IR Probe for the Electron Delocalization Study

Similar to the nitrile functionality, the stretching vibrational frequency of alkyne functionality ν(−C≡C−) and its corresponding molar extinction coefficient ε(−C≡C−) are responsive to electronic density across the triple bond. The examples of the −C≡C– stretching vibration used as an IR probe for charge dynamics are described in this section.

**Figure 7:**
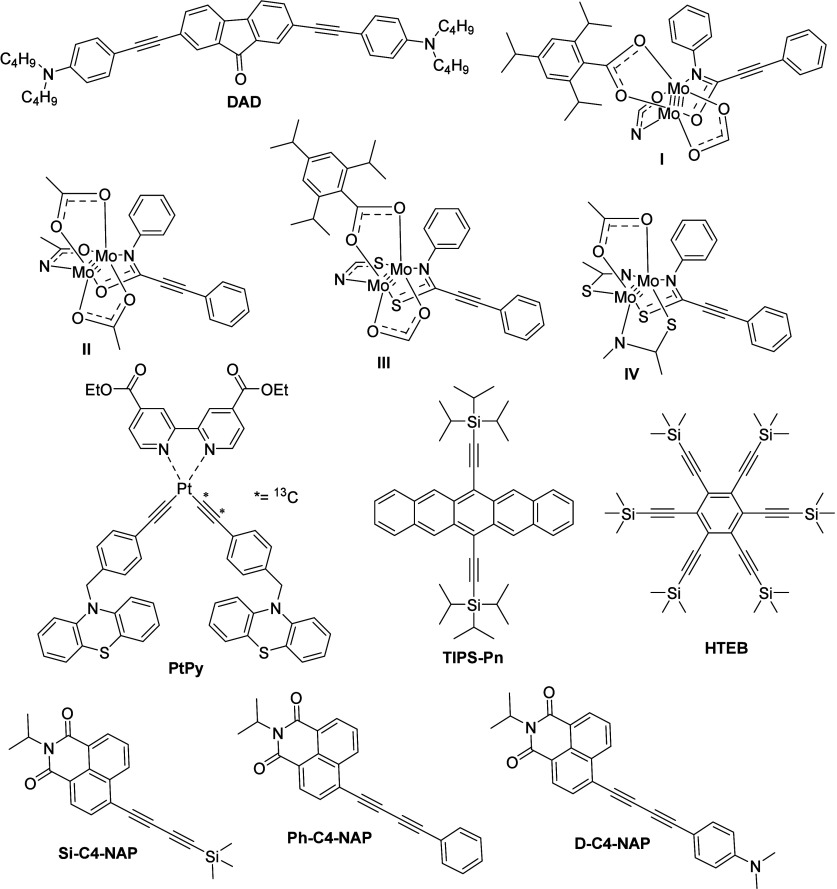
Structure of the molecules used for the electron delocalization study using the stretching vibration of the alkyne (−C≡C−) functionality.

**Figure 8:**
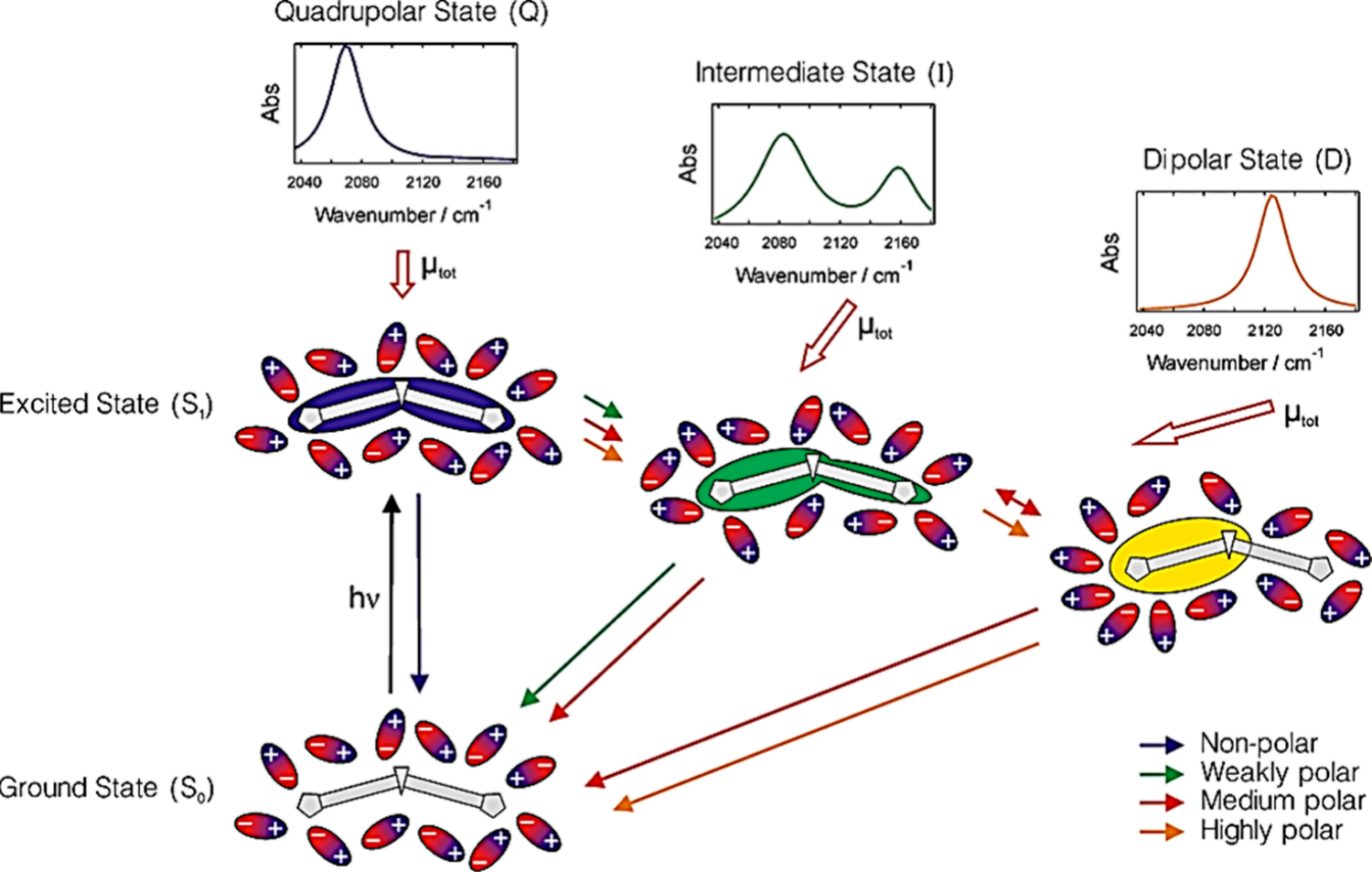
Schematic illustration of the nature of the **DAD** molecule’s lowest singlet excited state due to the effect of solvents of different polarities. The colored arrows denote the excited state pathways according to the polarity of the solvents. The total electric dipole moment is indicated by the empty arrows. The simulated spectra of IR absorption for the different excited states of the **DAD** molecule are based on the band shape analysis of the transient spectra in the stretch region corresponding to the −C≡C– functionality. Reproduced from ref (89). Copyright 2016 American Chemical Society.

Dereka et al.^89^ synthesized the quadrupolar D−π–A−π–D molecule (**DAD**, Figure 7) by referring to the previous literature,^108^ and the authors have used femtosecond TR-IR spectroscopy to study the symmetry-breaking dynamics in the synthesized molecule upon photoexcitation. The **DAD** molecule has an almost linear structure with a central fluorenone acceptor unit, which was attached to two branches comprising dibutylamine donor units linked through an acetylene bridge. Moreover, the **DAD** molecule exhibited several IR marker modes, for example, −C=C–, −C≡C–, and −C=O stretching vibrations, that can be probed to examine the excited state evolution in different parts of the **DAD** molecule. The authors preferred the −C≡C– stretching vibration attributed to its highly localized vibration and availability in both arms of the DAD molecule; also, its stretching frequency was well separated from those of the other modes. Their study found that the solvents of different dielectric properties play a decisive part in the extent of the symmetry-breaking process and its dynamics (Figure 8). Therefore, the authors stated that by selecting solvents with a suitable dielectric constant and relaxation dynamics, the emissive properties of this type of molecule can be adjusted even for larger systems such as conjugated polymers and dendrimers with symmetric D−π–A−π–D molecular architecture. This symmetry-breaking process leads to excitation energy concentration; therefore, this phenomenon can open new possibilities for applying this type of large multipolar conjugated molecule in light-harvesting devices. The understanding achieved here is also relevant for symmetric conjugated systems in rigid environments.

**Figure 9:**
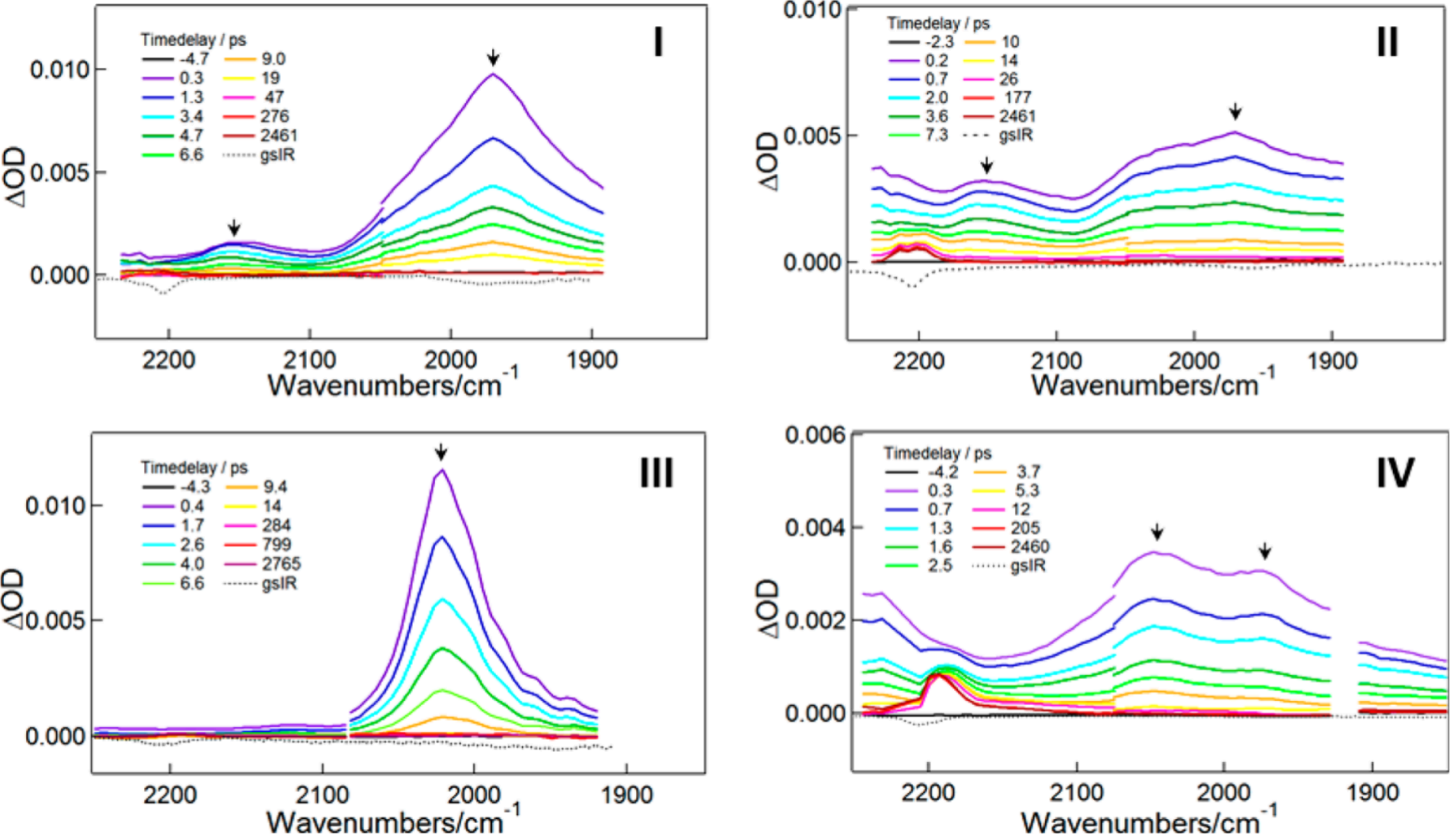
fs-TR-IR spectra of dimolybdenum paddlewheel molecules, **I**–**IV**, in the region of ν(−C≡C−), i.e., 1850–2250 cm^–1^. Dotted lines in the spectra depict inverted ground-state IR absorption spectra (gs-IR). Reproduced from ref (109). Copyright 2017 American Chemical Society.

In another study, Jiang et al.^109^ prepared the dimolybdenum paddlewheel molecules **I**–**IV** (Figure 7) to understand the interligand electron transfer and the role of the bridging units in it. The prepared molecules exhibited strong light absorption properties and were attributed to metal-to-ligand charge transfer (MLCT), i.e., charge transfer from molybdenum metal to the amide/thioamide-based ligands. In the fs-TR-IR spectra (Figure 9), especially for the ν(−C≡C−) bands, the authors noted the different distribution patterns on the ligands for the transferred electron in the ^1^MLCT S_1_ states of the prepared paddlewheel compounds. To evaluate the charge distribution in the MLCT excited states of the molecules, the authors have used a mixed-valence classification scheme, which is like the Robin–Day scheme^110,111^ (from class I to class III). The distribution of the charges in ^1^MLCT S_1_ states of amide-based compounds **I** and **II** was assigned as class II. Most of the transferred electron density was situated on one of the amide-based ligands; nonetheless, some of the electron density was also located on another amide-based ligand (*trans* to the first). However, delocalized transferred electron density was observed all over the two *trans*-thioamide ligands in the thioamide-based compound (**III**) in their ^1^MLCT S_1_ state and unequal distribution on the two *trans*-thioamide-based ligands in the ^1^MLCT S_1_ state of compound **IV**. Therefore, the authors assigned the ^1^MLCT S_1_ state of compound **III** as class III and compound **IV** as class II. The varying ν(−C≡C−) band patterns were observed by these authors for the prepared compounds, and it was ascribed to different rates of interligand electron transfer in their ^1^MLCT S_1_ state. Sulfur atoms in thioamide-based compounds appear to have strengthened the metal–ligand orbital mixing, which further encouraged interligand electron transfer and produced more delocalized ^1^MLCT S_1_ states. Their study also demonstrated that ligand configurations affect the mixing of metal–ligand orbital and interligand electron transport in the ^1^MLCT S_1_ states.

**Figure 10:**
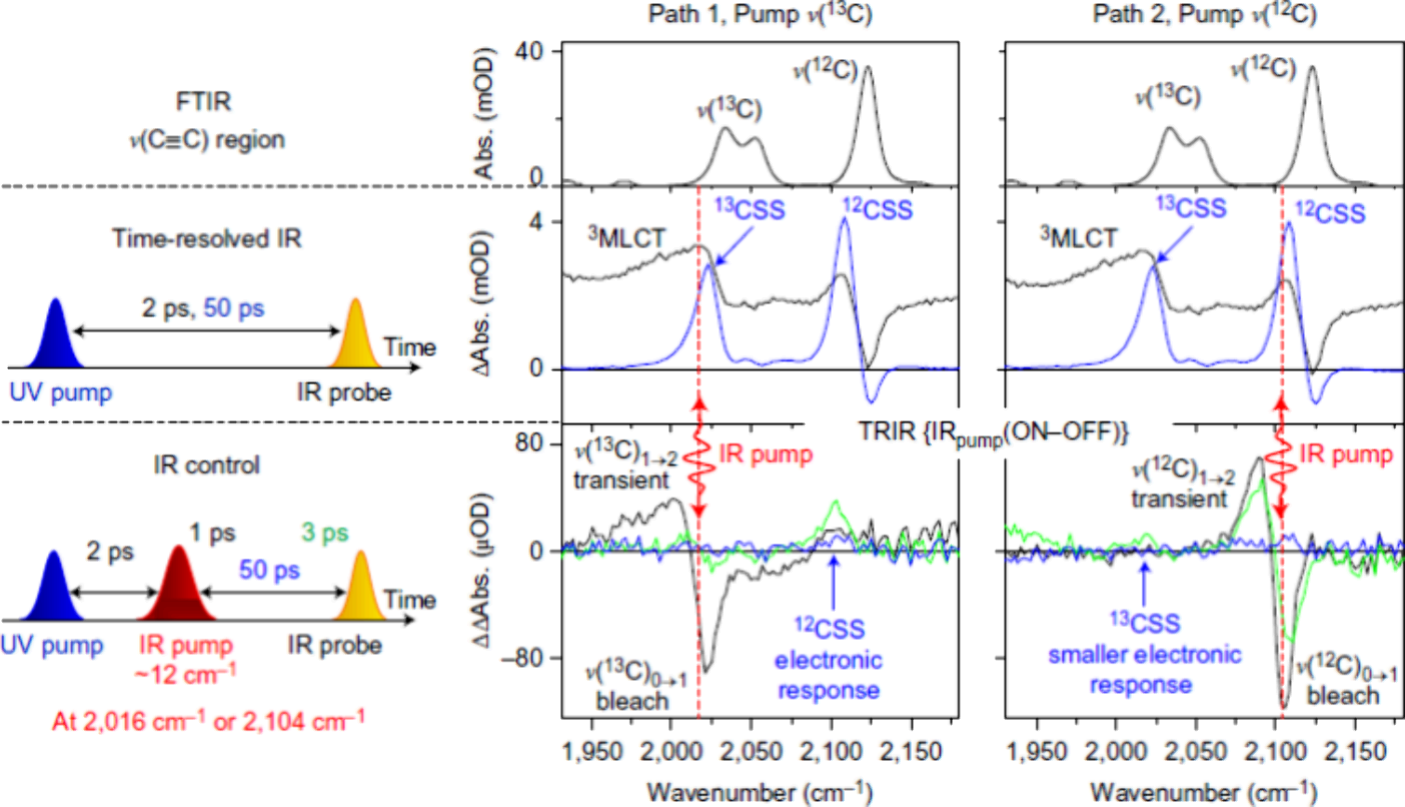
Summary of excited-state dynamics of the molecule (**PtPy**) in dichloromethane solvent with and without vibrational perturbation. Results for ν(^13^C) excitation are depicted in the middle panel, while results for ν(^12^C) excitation are shown in the right panel (2016 and 2104 cm^–1^, respectively). Reproduced with permission from ref (116). Copyright 2017 Springer Nature Limited.

Based on the new experimental techniques as well as groundbreaking theoretical research on vibrational control in molecular interferometers,^112−115^ Delor et al.^116^ prepared a fork-typed molecule (**PtPy**, Figure 7) comprising D–B–A–B–D backbone with two parallel, structurally distinct but electronically identifiable electron transfer pathways in order to achieve specific and targeted vibrational control over light-induced molecular function. Moreover, the authors substituted the ^13^C-isotope in one of the −C≡C– bridges in the molecule. This noninvasive alteration by the authors created a vibrational difference between the two electronically identical D→B→A pathways of electron transfer (Figure 10), which permitted selective excitation of one by mid-IR without changing the electronic behavior. By using this new molecular platform, the authors have employed the UV_pump_–IR_pump_–IR_probe_ pulse sequence to facilitate the transfer of electrons. In that sequence, the UV_pump_ initially establishes a charge transfer state and then transforms to either of the charges separated states (D^+•^–B–A^–•^) at equal rates; in Figure 10, it was designated as ^13^CSS or ^12^CSS based on their position on the ^13^C- or ^12^C-arm, respectively. Afterward, during electron transfer, the narrow-band IR_pump_ specifically stimulated bridge vibrational modes connected to one of the two competing electron transfer pathways. Ultimately, the IR_probe_ was employed by the authors to spectrally track the alterations in the CSS population brought about by the IR_pump_. Vibrational excitation unevenly disturbs the electron transfer possibilities at the excited state “crossroad” experienced by the system. Transient IR spectra with and without this intermediate vibrational excitation were recorded by the authors (Figure 10), and the IR_pump_ pulse was tuned to quantify the variation in the yield of CSSs caused by IR excitation. By using mid-infrared light, the authors were able to show active control over the spatial direction of intramolecular electron transfer. The authors’ discovery adds weight to the increasing body of evidence suggesting that molecular devices that rely on charge and energy transport could make reasonable use of vibronic coupling, which is a phenomenon that occurs frequently in nature.

Recently, Grieco et al.^117^ synthesized the bis(triisopropylsilylethynyl)pentacene (**TIPS-Pn**, Figure 7) molecule and examined the electronic state dynamics involved in singlet fission reaction using ultrafast mid-infrared (mid-IR) spectroscopy. During their study, they noted the time-dependent frequency corresponding to the alkyne stretch frequency of the triisopropylsilylethynyl side groups, which was connected to the conjugated pentacene core. Significantly, the authors have observed that line shape and frequency of the alkyne stretching mode vary with the temperature; therefore, it has resulted in distinctive vibrational frequencies for triplet excitons and hot ground-state molecules. These unique frequencies were further used to track the triplet pair separation dynamics throughout the singlet fission reaction. They were also able to measure relaxation processes that can compete with correlated triplet pair separation by tracking the energy dissipation into the molecular crystals on ultrafast time scales thanks to the temperature-dependent fluctuation in the alkyne stretching frequency of the **TIPS-Pn** molecule. From the ultrafast vibrational study, the authors found that the vibrational features were overlaid on a broad electronic transition. Therefore, in another study, Grieco et al.^118^ investigated the nature of this broad mid-IR electronic transition. Their study results revealed that this broad feature offers a probe for the correlated triplet pair intermediate dynamics, and was complementary to that of measurements for these states in the visible and near-IR regions of the spectra.^119−131^ Notably, the broad electronic transition was measured by the authors simultaneously with the vibrational stretching features of the electronic states that were involved in the singlet fission reaction. This study provided an independent probe for the dynamics of correlated triplet pairs that were compared quantitatively with the triplet state separation and relaxation routes discovered via vibrational modes.^117^

Moreover, by combining ultrafast visible and mid-IR transient absorption spectroscopy techniques, Grieco et al.^118^ revealed that the complete dissociation of correlated triplet pair intermediates to arise triplet excitons independently in **TIPS-Pn**-based crystalline films occurs on time scales from 100 ps to 1 ns ranges. This singlet fission reaction completion time scale was an order of magnitude longer than the previously obtained time scale for **TIPS-Pn** and other related derivatives.^122,123,132^ Their findings indicated that deactivation pathways of the excited state will compete with charge transfer or triplet harvesting^133,134^ in functional devices. Even in molecular systems like **TIPS-Pn**, where the initial singlet fission events occur on the picosecond time scale, this has to be managed on time scales significantly longer than previously anticipated to permit efficient harvesting of the multiplied triplet excitons.^135^

Very recently, using DFT calculations, Zhao and Wang^136^ studied the −C≡C– stretching vibrational properties in graphdiyne molecules with different molecular sizes. Their study revealed that as the molecular size of graphdiyne increases, one of the vibration intensities corresponding to −C≡C– stretching increases significantly because of a combined effect of vibrational delocalization and π-electron conjugation. Also, the study predicted that the −C≡C– stretching intensity can be enhanced more than 2000 times for the graphdiynic 46-mer than for the phenylacetylenic dimer. In their next study,^137^ they used the hexakis[(trimethylsilyl)ethynyl]benzene (**HTEB**, Figure 7) molecule to study the vibrational properties of the −C≡C– bond through conventional IR, the TR-IR, and the femtosecond 2D IR method. The authors observed the picosecond intramolecular energy redistribution process between two nondegenerate stretching modes of the −C≡C– bond. Moreover, the symmetry-breaking process was attributed to a halogen-bonding interaction between the **HTEB** molecule and solvent dichloromethane. From the 2D IR spectra, the authors noted the insignificant initial spectral diffusion value, which further suggested the limited structural dynamics and stiff structure of the **HTEB** molecules. Their research revealed the first nonlinear infrared study of the −C≡C– stretch, which is typically weak. The techniques described by the authors are crucial for gaining a thorough grasp of structure-related phenomena like vibrational energy transfer in novel molecules comprising −C≡C– functionality.

Li et al.^138^ synthesized butandiyn-diyl-bridge-containing molecules (**Si–C4–NAP**, **Ph–C4-NAP**, and **D-C4-NAP**), and the structures of the molecules are depicted in Figure 7. Femtosecond-nanosecond transient absorption analysis in the visible and mid-IR regions following 400 nm excitation was performed by the authors for those synthesized molecules in toluene and dichloromethane solvents. Results revealed that transient mid-IR spectra corresponding to the stretching modes of the −C≡C– bridge play a crucial role in understanding the excited state electronic dynamics. Also, highly polarized/charge-separated state formation was noted for the **D-C4-NAP** molecule. In addition, they tracked the vibronic relaxation, dihedral angle equilibration, triplet state formation, charge separation, and charge recombination processes in the excited electronic states. Furthermore, time-dependent DFT examination of the molecules in their normal mode and excited electronic states enabled the authors to assign the observed dynamics to the specific electronic transitions. The authors have demonstrated that the torsional barrier to rotation in the molecular ground state is less than *k*_B_*T*, which further provides access to a wide range of dihedral angles. The dihedral angle influenced the excited state dynamics, including charge separation. It is interesting to note that the torsional motion and corresponding changes in spectroscopic data enabled the examination of the electron transfer dynamics associated with molecular subpopulations.

The −C≡C– stretching vibration has gained significance in the field of energy science, remarkably for studying dynamics of the charge separation process and energy transfer in OSCs, graphyne, graphdiyne, and other related molecules. The −C≡C– bond in the organic molecules serves as a conjugation bridge. Moreover, the −C≡C– stretching frequency is much less explored as an IR reporter when compared to the −C≡N stretching vibration, owing to their lower molar absorptivity. However, Kossowska et al.^139^ showed significant enhancement in ε(−C≡C−) of an alkyne functionality by substituting a Si atom to the alkyne group of interest. In addition to the vibrational frequency, its molar extinction coefficient is sensitive to the electron density distribution.

### 3.2 The Study Outputs

- TR-IR spectroscopy is used to study the vibrational modes in symmetric molecules with a D−π–A−π–D backbone, which provides comprehension into charge transfer mechanisms and excited-state symmetry breaking.
- By using ultrafast mid-IR spectroscopy, dynamics in singlet fission processes were probed, especially the triplet exciton separation and correlated triplet pair intermediates.
- Some research studies were focused on isotopic labeling in the molecules to understand electron transfer pathways, which showcased the vibrational mode manipulations to influence charge dynamics.
- Study on D−π–A−π–D-based molecules showcased how solvent polarity affects symmetry breaking and electron density concentration, which is very important for light-harvesting applications.
- For **TIPS-Pn** molecules, the authors could track triplet pairs and their dynamics, which helps in understanding the behavior of exciton postsinglet fission.
- The π–π conjugation in the molecule and solvent interactions enhances −C≡C– stretching absorption bands, especially in **HTEB** molecules, which implies its utility in structural dynamic study as well as in studying energy flow in two-dimensional materials.
- Studies suggested the tuning of solvent interactions with the molecule by varying polarity can optimize emissive states of the molecules in multipolar systems, which can further enhance energy capture ability and their conversion.
- By selective vibrational excitation, the direction of the charge flow can be controlled, which indicates that optimized energy transport can be attained in the molecular devices by manipulating vibronic coupling potential.

## 4 Molecules with Carbonyl (−C=O) Functionality

The −C=O functionality is an organic moiety with a double bond between carbon and oxygen atoms.^140^ Furthermore, the −C=O moiety could form a bond with other functional groups to attain the numerous carbonyl-bearing functionalities, for example, ketones, aldehydes, carboxylic acids, and their derivatives.^141^ Moreover, the compounds bearing carbonyl functionality can be classified into three types depending upon the molecular structure: small molecules, polymers, and organic salts.^142^ The −C=O group’s bond length is widely known to be 1.22 Å. Since the oxygen atom has a higher electronegativity (3.5) than the carbon atom (2.5), the electron cloud dispersion can flow in the direction of the oxygen atom. This property is exciting because it induces the polarity in the carbonyl group and activates their chemical reactivity.^143^ In addition, in most of the D–A-type conjugated small/polymeric molecules,^144,145^ carbonyl functionalities are often found in electron-accepting cores such as cyanopyridones,^146^ diketopyrrolopyrrole,^147^ and naphthalene diimide.^148^ Besides, these carbonyl functionalities can be used as IR reporters to investigate the behavior and nature of the charges in those organic molecules. Carbonyl vibrations have a behavior akin to that of the solitary diatom (CO), mainly decoupled from the other parts of the molecule.^53,149−152^ Furthermore, it has been demonstrated that carbonyl probes are the most reliable IR reporters for neutral compounds.^53^

### 4.1 –C=O Functionalities as the IR Probe for the Electron Delocalization Study

The examples of the −C=O stretching vibration used as an IR probe for charge dynamics are described in this section.

**Figure 11:**
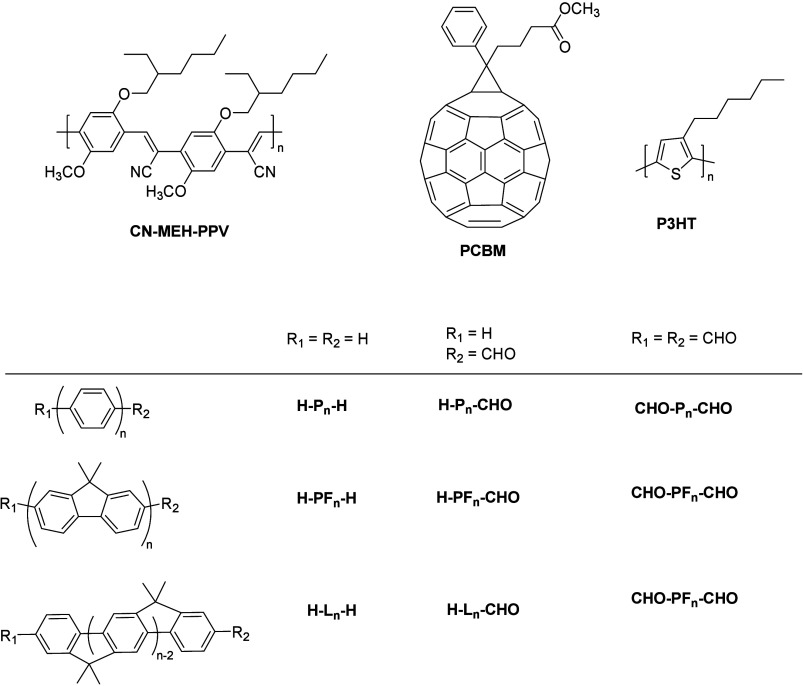
Structure of the molecules used by Asbury et al.^156−159^ and Talipov et al.^164^

**Figure 12:**
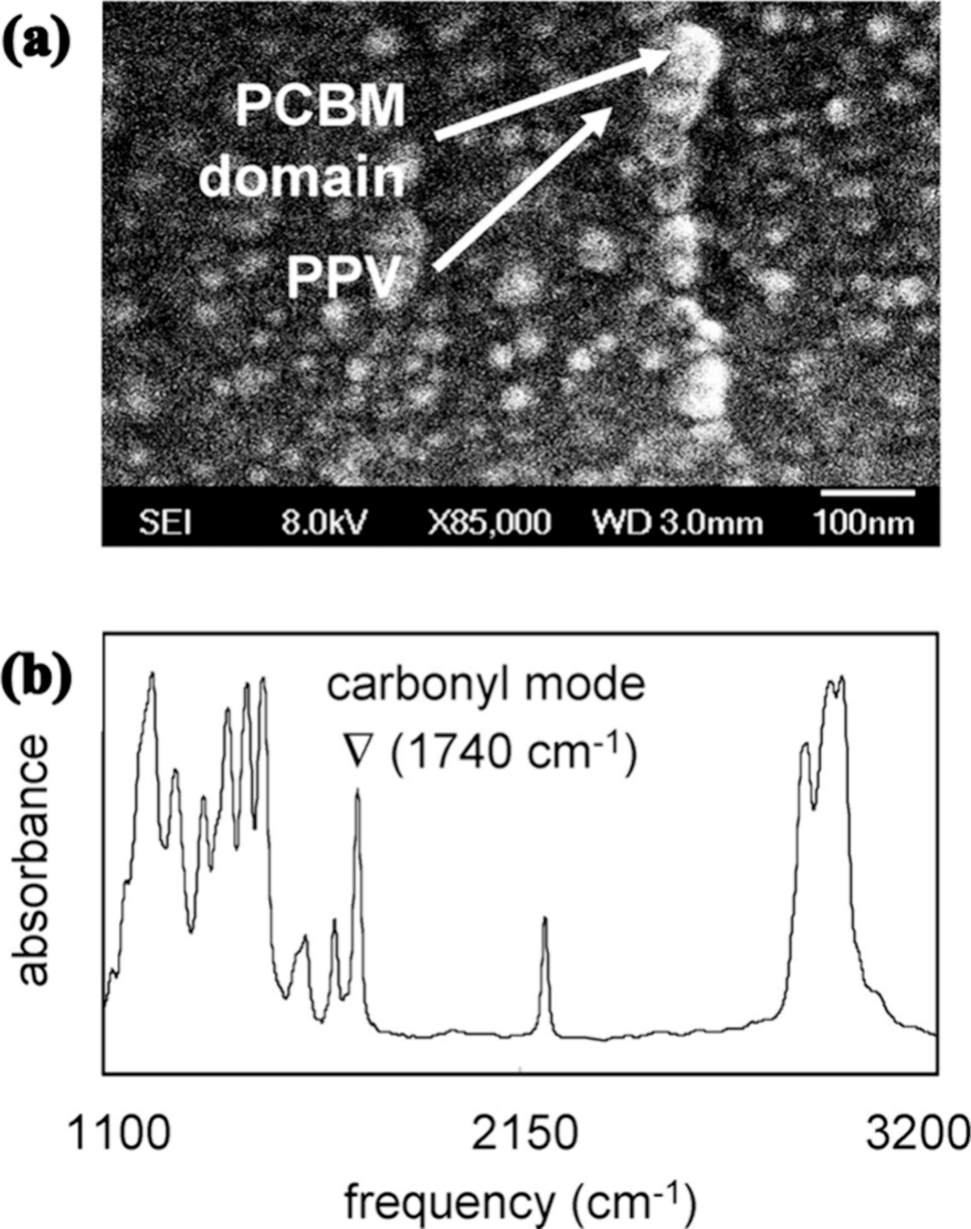
(a) Morphology of the polymer blend, i.e., **CN-MEH-PPV:PCBM**. (b) IR absorption spectrum of the **CN-MEH-PPV:PCBM**. Reproduced from ref (159). Copyright 2007, American Chemical Society.

To study bulk morphological changes, IR spectroscopy has been used in several studies.^153−155^ 2D IR spectroscopy has been used by Asbury et al. to examine the homogeneity of organic PV molecule, i.e., **PCBM** (Figure 11) in thin films, involving the polymers **P3HT** and **CN-MEH-PPV** (Figure 11).^156−159^ Moreover, the authors used the combination of 2D IR and visible pump–infrared (Vis–IR) probe spectroscopy to investigate the **PCBM** at the molecular level to identify and study the reasons for the low charge carrier mobility in OSCs.^159^ For the study, the authors prepared a polymer blend using conjugated polymer (**CN-MEH-PPV**) and functionalized fullerene with electron-accepting units (**PCBM**). The morphology of the prepared polymer blend was studied using scanning electron microscopic analysis, and it revealed the spherical morphology of the **PCBM** molecule that was surrounded by the **CN-MEH-PPV** polymer (Figure 12a). To examine the structure and charge dynamics of the prepared system, the authors utilized the carbonyl stretching frequency of the fullerene molecule, and the band corresponding to that appeared at a frequency of 1740 cm^–1^ (Figure 12b). From the 2D IR and Vis–IR analysis, the authors found a correlation between the stretching frequency of the carbonyl functionality attached to the fullerene molecule and their radial position. In addition, the authors used this correlation to calculate the electron’s average radial velocity as they transfer from the interfaces to the center part of the roughly spherical fullerene domains. From this, the authors have noted the radial velocity of 1–2 m/s, which suggests that the fullerene molecule alone has higher intrinsic electron mobility than fullerene domains in the composite polymer blend. Furthermore, they observed the constant radial velocity even at different temperatures, which points out that the formation of free charge carriers in the prepared blend **CN-MEH-PPV:PCBM** occurs on an activation-less pathway.^160−163^ Finally, their study suggests that the PCE of OSCs can be further improved if one can control the phase separation to reduce the interfacial boundaries along the migration paths of the charge.

By combining DFT and a multistate parabolic model (MPM), Talipov et al.^164^ analyzed the polaron distribution in nine conjugated oligomer series (Figure 11). For oligomeric molecules without a carbonyl moiety, the authors have noticed a significant consistency in the distribution of charges, where the charge was found predominantly in the middle part of the molecule, involving 7–8 units. Additional extension of the polaron was impeded by the reorganization of the structure/solvent and associated reorganization energy. In the asymmetric oligomers, i.e., oligomers with mono carbonyl functionality, the authors observed a shift of the polaron toward the end of molecules where carbonyl moiety attached. Remarkably, the oligomers bearing two carbonyl functionalities displayed a more complex behavior. At first, the authors observed the spanning of the polaron distribution across both terminal units. But, as the number of internal repeating units increased, it exhibited a tendency to localize polaron toward one end, which is similar to the behavior of polaron in asymmetrically capped systems, i.e., oligomers bearing mono carbonyl unit. Moreover, the DFT data for the carbonyl vibration indicated that the IR-shift, which was triggered by one-electron reduction of the oligomeric molecules, displayed faster convergence to the polymeric limit than the progression of electronic structural/geometrical limits. This examination is crucial in the circumstances in which the vibration of the carbonyl moiety is readily observed using IR spectroscopic analysis, and thus it could be employed to experimentally probe the charge delocalization. Since the charge carried by the carbonyl functional group reaches the polymeric limit faster when compared to the polaron delocalization on conjugated repeating parts of the oligomers. This factor can be considered while doing the interpretation of the experimental data.

The usage of the −C=O functionality to probe charges and excitons has remained largely underexplored.

### 4.2 The Study Outputs

Most research papers investigated the charge separation and trapping dynamics in **CN-MEH-PPV:PCBM** blends by ultrafast vibrational spectroscopy. Key findings of this section include:

- The previously studied results depict that upon photoexcitation of the prepared **CN-MEH-PPV:PCBM** blends, the charge transfer can be observed at the interfaces between the blend, with processes spanning from around 100 fs to ns.
- The stretching frequency of carbonyl (−C=O) functionality in the **PCBM** molecules serves as a sensitive probe for studying charge transfer mechanisms, reflecting the **PCBM**’s proximity to the interfaces of the prepared blend and signifying the formation of charge-separated states by dissociation of charge transfer states.
- An increase in the temperature helps the electrons to escape from charge transfer states, which further enhances the formation of charge separation. Moreover, over longer time scales, the results also depicted the entrance of electrons to shallow trap states within clusters of **PCBM** molecules.
- Literature also depicts the slower charge transfer component and can be attributed to the need for excitations in the prepared conjugated polymer systems to diffuse toward the interfaces of the blend, which results in a 3 ns time constant for this process.
- The literature emphasizes that the stretch frequency of the carbonyl functionality varies based on the local environment around the **PCBM** molecules. The higher frequency bands indicate **PCBM**’s proximity to polymer interfaces. Moreover, this gradient facilitates the elucidation of the charge photogeneration dynamics and the phase separation effects in the materials.

Largely, these literature findings suggest a relationship between the ultrafast charge transfer dynamics and the stretching vibration of the carbonyl moiety. Moreover, the findings also depict the effect of temperature and phase separation, with suggestions for improving the PCE of OPV materials.

## 5 Conclusion and Perspectives

IR spectroscopy is rapid, sensitive, and nondestructive in the chemical fingerprinting of various materials. The collected spectroscopic data of the organic molecules delivers distinctive insights by directly observing vibrational dynamics in real-time, which further offers clear information about the molecular structure on ultrafast time scales. The results of time-resolved electronic methods (absorption and emission) usually smear out the vibrational details because of the coupling between electronic states and the solvent used for the study. The results obtained from TR-IR analysis help us to understand the vibrational energy transport mechanism and how it is coupled to electronic processes. Moreover, this method allows the exploration of interactions between the molecules and their local surroundings, delivering information about how solvents influence the flow of vibrational energy. TR-IR analysis helps to understand complex absorption/emission spectra and excited-state chemical processes in energy applications like photocatalysis and solar cells, where vibrational dynamic aspects play a very important role in device performance. However, the integration of TR-IR methods with other transient methods is a powerful means of understanding electronic processes in material systems.

Currently, most of the research in energy science emphasizes enhancing the performance of optoelectronics, typically by using a conjugated small/polymeric molecule. The stretching vibration of the functional groups serves as a significant tool for charge transfer assessment in these organic molecules, with their stretching frequency and their molar extinction coefficient related to the total charge on the functionality. Therefore, this review article collectively underscores the usage of vibrational spectroscopy for studying the complexities of charge transfer dynamics in various molecular architectures comprising IR reporter functionalities like nitrile (−C≡N), alkyne (−C≡C−), or carbonyl (−C=O). Most of the research has proven a linear relationship between stretching frequency of −C≡N functionality and the charge, which allowed the quantification of charge separation in the molecules. Furthermore, by observing the variation in the molar extinction coefficient with the charge state, one can analyze the charge dynamics. By observing the variation in localized vibrational modes of the functional groups, researchers can understand the excited-state processes and their dynamics, paving the way for advancements in the design of energy materials and their applications. Most importantly, in comparison to the −C≡N stretching vibration, the −C≡C– and −C=O stretching vibrations are less utilized as an IR reporter to examine charge transfer dynamics, owing to their lower molar absorptivity. Nevertheless, it can be significantly enhanced by modifying the structure, for instance, attaching the Si atom to the alkyne functionality, which increases the molar absorptivity of the alkyne functionality.

The IR approach has been proven to be a unique method to obtain information regarding the electron density in molecules. However, related studies are still limited, and more theoretical and experimental work is compulsory to fully understand how it applies to other conjugated molecules comprising −C≡N, −C≡C–, and −C=O moieties, which have shown good performances in energy conversion and storage devices. For example, many molecules with these functional groups gave good efficiencies in dye-sensitized solar cells.^16,165^ They were good passivators in PSCs by promoting electron transport to the cathode,^166^ were good hole transport materials in PSCs,^167,168^ and were also found in energy storage applications as electrodes, electrolyte additives, and separators.^169^

Although this method of studying electron density in molecules is still in the early stages, in the near future, we expect it to have significant potential for improvement. As research progresses, there will be advancements in the exploration of this technique in various materials which are expected to add to the performance of energy conversion and storage devices, and it can give the relationship between structure and device performance. As of now, this technique has only begun to uncover a few of the possibilities.

During the past decade, research and development of electron delocalization in energy conversion and storage has made great strides.^29,31,170,171^ Despite the progress, many challenges and opportunities remain with the move from molecular properties to devices. The following paragraph briefly summarizes our viewpoints.

- The rational design of molecules represents the top priority to precisely control the extent of electron delocalization in molecules and materials for specific applications. It is well-accepted that the rigid and coplanar conformations of conjugated molecules^28,172,173^ exhibit distinct electron delocalization characteristics. For example, ladder-type, oligo-, and poly(*p*-phenylene)s^174^ offer an excellent model system to study electron delocalization and electron mobility along a coplanar conjugated backbone. Such conjugated molecules usually have very limited solubility in organic solvents,^28^ limiting their solution processability. This issue can be addressed by introducing solubilizing groups (e.g., alkyl chains) to the bridgeheads.^175^ Given the synthetic challenges, DFT^176−179^ calculations and machine learning^8,180−185^ would help screen and optimize the molecular structures before their laboratory syntheses.
- Controlling material morphology (e.g., the active layer of bulk heterojunction OSCs) is crucial for efficient charge generation and carrier transport.^186,187^ High-resolution atomic force microscopy^188^ and resonant soft X-ray scattering^189^ are well suited to study the nanostructures and establish the relationship between morphology and energy conversion and storage performance.
- If the benchwork is successful, scaling up controlled syntheses and device assembly, testing, and deployment should be explored, together with life cycle assessment^190^ and techno-economic analysis,^191^ for possible potential commercialization.

The main intention of this review article is to provide a systematic summary of the existing works of literature and their results, which might inspire new ideas as well as serve as a foundation for future study. The tactics and study findings across this literature help researchers to understand how molecular interactions can be engineered to obtain increased performance in energy conversion and storage devices.
